# Astilbin Inhibits High Glucose-Induced Inflammation and Extracellular Matrix Accumulation by Suppressing the TLR4/MyD88/NF-κB Pathway in Rat Glomerular Mesangial Cells

**DOI:** 10.3389/fphar.2018.01187

**Published:** 2018-10-18

**Authors:** Fang Chen, Xiaoguang Zhu, Zhiqiang Sun, Yali Ma

**Affiliations:** Department of Nephrology, Huaihe Hospital of Henan University, Kaifeng, China

**Keywords:** diabetic nephropathy, inflammation, extracellular matrix accumulation, astilbin, TLR4/MyD88/NF-κB pathway

## Abstract

Diabetic nephropathy (DN) is characterized by inflammatory responses and extracellular matrix (ECM) accumulation. Astilbin is an active natural compound and possesses anti-inflammatory activity. The aim of this study was to evaluate the anti-inflammatory effect of astilbin on high glucose (HG)-induced glomerular mesangial cells and the potential mechanisms. The results showed that HG induced cell proliferation of HBZY-1 cells in a time-dependent manner, and astilbin inhibited HG-induced cell proliferation. The expression and secretion of inflammatory cytokines, including interleukin-6 (IL-6) and tumor necrosis factor alpha (TNF-α), and ECM components, including collagen IV (Col IV) and fibronectin (FN), were induced by HG. Moreover, TGF-β1 and CTGF were also induced by HG. The induction by HG on inflammatory response and ECM accumulation was inhibited after astilbin treatment. Astilbin treatment also attenuated HG-induced decrease in expression of matrix metalloproteinase (MMP)-2 and MMP-9. The TLR4/MyD88/NF-κB pathway was activated by HG, and the inhibitor of TLR4 exhibited the same effect to astilbin on reversing the induction of HG. TLR4 overexpression attenuated the effect of astilbin on HG-induced inflammatory cytokine production and ECM accumulation. The results suggested that astilbin attenuated inflammation and ECM accumulation in HG-induced rat glomerular mesangial cells via inhibiting the TLR4/MyD88/NF-κB pathway. This work provided evidence that astilbin can be considered as a potential candidate for DN therapy.

## Introduction

Diabetic nephropathy (DN) is a kind of kidney disease that affects approximately 25% of the patients with type 2 diabetes (Lytvyn et al., 2016; Tesch, 2017). DN is one of the most common causes of end-stage renal disease (ESRD), and the patients with ESRD often require hemodialysis or even kidney transplantation to recover the kidney function (Tang et al., 2015). There are a variety of risk factors that promote the development and progression of DN, including long duration of diabetes, elevated glucose levels, high blood pressure, and dyslipidemia (Tziomalos and Athyros, 2015).

Increasing studies indicate that identification and management of risk factors for DN is of paramount importance (Tziomalos and Athyros, 2015). Elevated glucose level is one of the main risk factors for the development and progression of DN (Tziomalos and Athyros, 2015). High blood sugar may lead to the formation of advanced glycation end products, which induces inflammation in the kidney and promotes the development of DN (Tziomalos and Athyros, 2015). Besides, excessive accumulation and deposition of extracellular matrix (ECM) is the major pathological alteration in DN, which results in the expansion of mesangial matrix, thickening of glomerular basement membrane and tubulointerstitial fibrosis (Dugbartey, 2017). Therefore, inhibiting inflammation and ECM accumulation is important for the management of DN.

**FIGURE 1:**
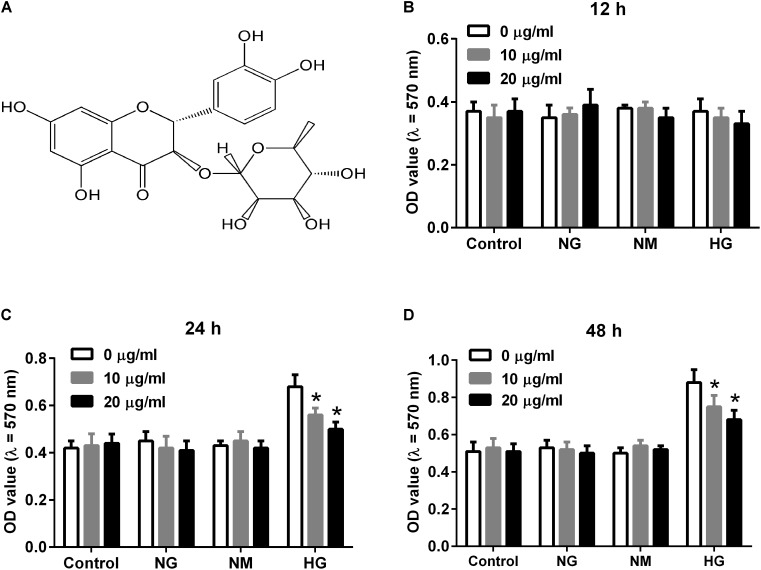
Effect of astilbin on HG-induced proliferation of HBZY-1 cells. Cells were cultured under normal glucose condition (NG, 5.5 mM D-glucose), mannitol condition (NM, 5.5 mM D-glucose + 24.5 mM mannitol), or high glucose condition (HG, 30 mM D-glucose) in the presence of astilbin (0, 10, and 20 μg/ml) for 12, 24, or 48 h. **(A)** Chemical structure of astilbin. Cell proliferation was detected using MTT assay after incubation for 12 **(B)**, 24 **(C)**, and 48 h **(D)**. ^∗^*P* < 0.05 vs. HG-stimulated group (without astilbin). Statistical significance was determined by one-way ANOVA.

Astilbin (**Figure 1A**) is an active natural compound belonged to flavonoid (Huang and Liaw, 2017). It is isolated from many kinds of herbs such as the rhizome of *Smilax china* L. (Smilaceae), and has been reported to possess various activities including anti-inflammatory and immunoregulatory effects (Meng et al., 2016; Yu et al., 2017). Recently, astilbin is reported to protect mice from kidney injury via regulating oxidative stress and inflammatory response in an *in vivo* study (Wang et al., 2016). However, the role of astilbin in DN remains unknown.

In the present study, the role of astilbin in high glucose (HG)-induced glomerular mesangial cells and the mechanism were investigated. We found that astilbin attenuated HG-induced inflammatory responses and ECM accumulation by inhibiting the TLR4/MyD88/NF-κB pathway. This work provided evidence that astilbin could be considered as a new candidate for DN therapy.

## Materials and Methods

### Cell Culture and Transfection

Rat glomerular mesangial cell line (HBZY-1) was obtained from Boster Biological Technology Co., Ltd. (Wuhan, China) and maintained in Dulbecco’s modified Eagle’s medium (DMEM) containing 10% fetal bovine serum (FBS) at 37°C. The TLR4 overexpression plasmid pcDNA3.1-TLR4 and empty pcDNA3.1 vector were purchased from GenePharma (Shanghai, China). Transfection of pcDNA3.1-TLR4 and pcDNA3.1 vector was performed using Lipofectamine 2000 (Invitrogen, Carlsbad, CA, United States) according to the manufacturer’s instructions. At 48 h after transfection, western blot was performed to determine transfection efficiency.

### MTT Assay

HBZY-1 cells were seeded into a 96-well plate with the density of 1 × 10^4^ cells/well. Cells were cultured under normal glucose condition (NG, 5.5 mM D-glucose), mannitol condition (NM, 5.5 mM D-glucose + 24.5 mM mannitol), or high glucose condition (HG, 30 mM D-glucose) in the presence of 10 and 20 μg/ml astilbin (purity >98%, Tauto Biotech Co., Ltd., Shanghai, China) for 12, 24, or 48 h. The concentrations of astilbin used were based on our previous study (Chen et al., 2018). Cells were then incubated with 100 μl MTT solution (0.5 mg/ml) for 4 h at 37°C. Subsequently, DMSO was added to dissolve the purple crystal and the absorbance was measured at 570 nm using a microplate reader (Bio-Rad Laboratories, Inc., Hercules, CA, United States). Each experiment was performed three times in triplicate.

### Real-Time RT-PCR

Total RNA was extracted from cells with different treatments using TRIzol Reagent (Invitrogen), and the concentration of the RNA was determined spectrophotometrically at the wavelength of 260 nm. cDNA was generated using total RNA (3 μg) with Superscript II reverse transcriptase with oligo (dT) primers (TOYOBO Life Science, Osaka, Japan). Finally, RT-PCR was carried out using SYBR^®^ Premix Ex Taq^TM^ II (Takara) according to the manufacturer’s instruction. GAPDH was used as an internal standard for semiquantitative analysis of the PCR amplification. The results were quantified by using the 2^-ΔΔCt^ method. The primers used were: interleukin-6 (IL-6) sense 5′-TCCA GCCA GTTG CCTT CTTG-3, anti-sense 5′-AGCC ACTC CTTC TGTG ACTC-3′; tumor necrosis factor alpha (TNF-α) sense 5′-CCAG AACT CCAG GCGG TGTC-3′, 5′-GGCT ACGG GCTT GTCA CTCG-3′; collagen IV (Col IV) sense 5′-ATTG GTGG CTCT CCAG GAAT CACA G-3′; anti-sense 5′-GGTG GTCC GGGG CTAC CCAA CGGT-3′; fibronectin (FN) sense 5′-TTAC CCTT CCAC ACCC CAAT CT-3′, anti-sense: 5’-TACA TTCG GCAG GTAT GGTC TTG-3′; transforming growth factor β1 (TGF-β1) sense 5′-GGCG GTGC TCGC TTTG TA-3′, 5′-TCCC GAAT GTCT GACG TATT GA-3′; connective tissue growth factor (CTGF) sense: 5′-GCTG GAGA AGCA GAGT CGTC-3′, anti-sense: 5′-ACAC CCCA CAGA ACTT AGCC-3′; GAPDH sense 5′-GGCA AGTT CAAC GGCA CAGT-3′, anti-sense 5′-ATGA CATA CTCA GCAC CGGC-3′. Each experiment was repeated three independent times.

### Enzyme-Linked Immunosorbent Assay (ELISA)

The supernatant of HBZY-1 cells after different treatments was collected, the contents of IL-6, TNF-α, Col IV, FN, TGF-β1, and CTGF were measured by ELISA using commercial kits (CUSABIO, Wuhan, China) in accordance with the manufacturer’s instructions. Each experiment was performed three times in triplicate.

### Western Blot

Cell extracts were prepared using the radio immunoprecipitation assay (RIPA) buffer. Then the protein concentration of the cell extracts was measured using a BCA protein assay kit (Pierce Biotechnology, Rockford, IL, United States). The protein samples were separated by 10% SDS–PAGE and transferred onto PVDF membrane. After blocking with 5% (w/v) non-fat milk for 1 h at 37°C, the membrane was incubated with primary antibodies against TLR4, MyD88, p-p65, p65, matrix metalloproteinase (MMP)-2, MMP-9, and β-actin (Abcam, Cambridge, MA, United States) at 4°C overnight. Then the proteins were probed by incubating with HRP-conjugated secondary antibody for 2 h at 37°C. Finally, the brands were detected using ECL reagent (Millipore, Billerica, MA, United States). Each experiment was repeated three independent times.

### Statistical Analysis

Data are expressed as mean ± SD. Comparisons between groups were performed using one-way ANOVA for multiple comparisons and the Student’s *t-*test for two groups. Statistical analysis was performed using SPSS version 13.0 (SPSS, Chicago, IL, United States). A difference was considered significant when *p*-value was less than 0.05.

## Results

### Astilbin Inhibited HG-Induced Proliferation of HBZY-1 Cells

To evaluate the effect of astilbin on HG-induced proliferation of HBZY-1 cells, cells were induced with NG, NM or HG in the presence of astilbin (0, 10, and 20 μg/ml) for 12, 24, and 48 h. The results in **Figure 1B** showed that HG and astilbin did not affect the proliferation of HBZY-1 cells with the incubation time of 12 h. However, after incubation for 24 or 48 h, the proliferation of HBZY-1 cells was significantly induced by HG, indicating that HG induced cell proliferation in a time-dependent manner. The induction was inhibited by astilbin (**Figures 1C,D**). Based on these results and a previous study (Wang et al., 2017), 24 h was selected as treatment time in the following experiments.

### Astilbin Decreased Inflammatory Cytokine Production Under HG Condition

**FIGURE 2:**
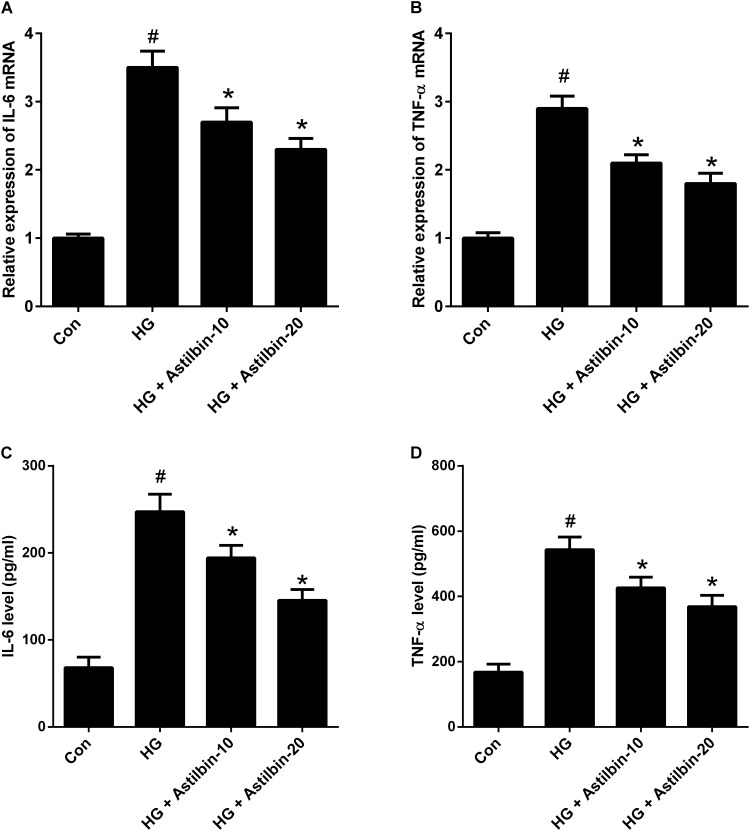
Effect of astilbin on HG-induced inflammatory cytokine production. HBZY-1 cells were treated with 10 and 20 μg/ml of astilbin under HG condition for 24 h. The mRNA levels of IL-6 **(A)** and TNF-α **(B)** in HBZY-1 cells were detected by qRT-PCR. The secretion levels of IL-6 **(C)** and TNF-α **(D)** in cell supernatant were detected by ELISA. ^#^*P* < 0.05 vs. Con (control group). ^∗^*P* < 0.05 vs. HG-stimulated group. Statistical significance was determined by one-way ANOVA.

It has been reported that HG could induce inflammation responses, thus the mRNA and protein levels of IL-6 and TNF-α were detected by qRT-PCR and ELISA. As shown in **Figures 2A,B**, HG induced the mRNA levels of IL-6 and TNF-α in HBZY-1 cells, while astilbin (10 and 20 μg/ml) attenuated the induction. In addition, the contents of IL-6 and TNF-α in cell supernatant were increased under HG condition, and the increase was inhibited by astilbin treatment (**Figures 2C,D**).

### Astilbin Suppressed HG-Induced ECM Accumulation

**FIGURE 3:**
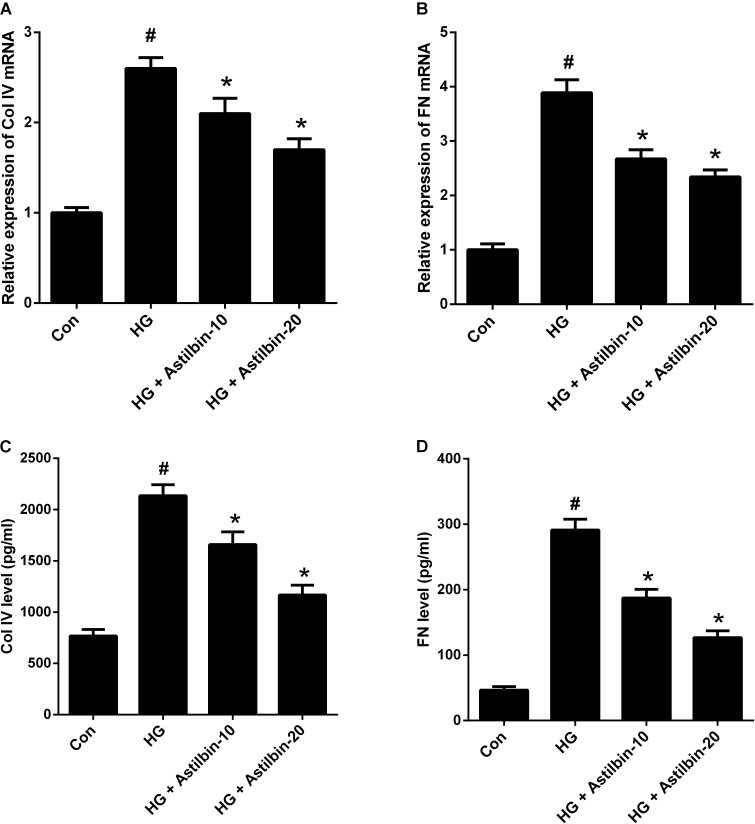
Effect of astilbin on HG-induced ECM accumulation. HBZY-1 cells were treated with 10 and 20 μg/ml of astilbin under HG condition for 24 h. The mRNA levels of Col IV **(A)** and FN **(B)** in HBZY-1 cells were detected by qRT-PCR. The secretion levels of Col IV **(C)** and FN **(D)** in cell supernatant were detected by ELISA. ^#^*P* < 0.05 vs. Con (control group). ^∗^*P* < 0.05 vs. HG-stimulated group. Statistical significance was determined by one-way ANOVA.

ECM accumulation is an important pathological change of DN and plays a crucial role in the development of renal fibrosis (Kolset et al., 2012). As shown in **Figures 3A,B**, the mRNA levels of Col IV and FN were increased in HBZY-1 cells under HG condition. Treatment with astilbin attenuated the effect of HG on the mRNA levels of Col IV and FN. Besides, HG induced the secretion of Col IV and FN in the cell supernatant, while astilbin treatment inhibited the induction (**Figures 3C,D**).

### Astilbin Inhibited the Expression of TGF-β1, CTGF, and MMPs

**FIGURE 4:**
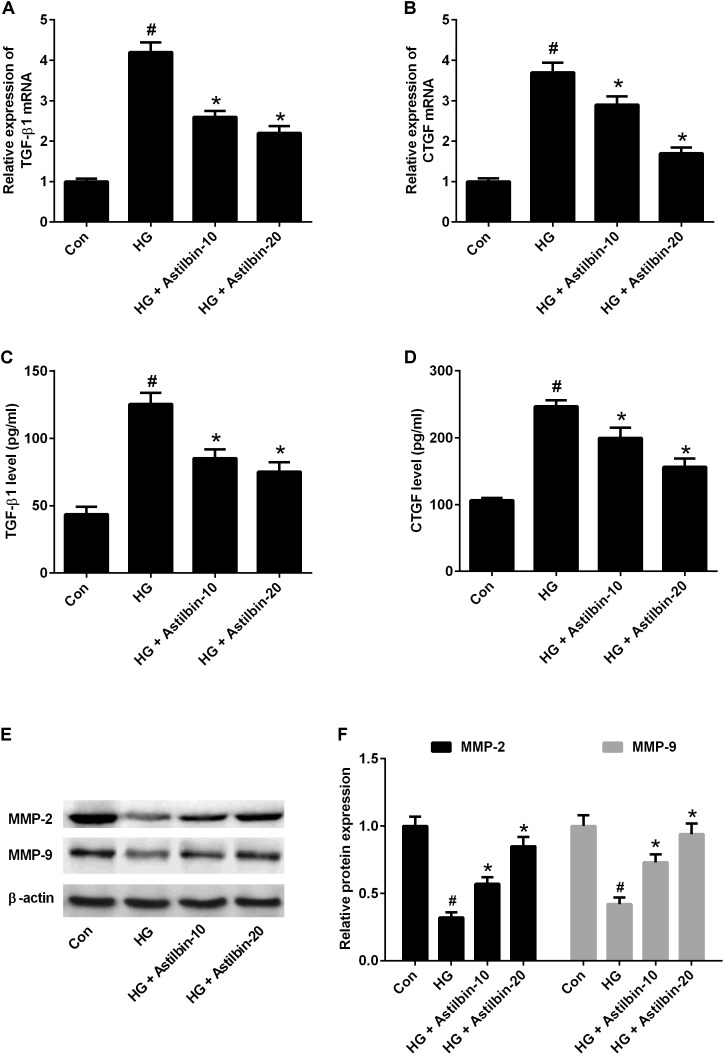
Effect of astilbin on HG-induced the expression of TGF-β1, CTGF, MMP-2, and MMP-9. HBZY-1 cells were treated with 10 and 20 μg/ml of astilbin under HG condition for 24 h. The mRNA levels of TGF-β1 **(A)** and CTGF **(B)** in HBZY-1 cells were detected by qRT-PCR. The secretion levels of TGF-β1 **(C)** and CTGF **(D)** in cell supernatant were detected by ELISA. **(E)** The expression levels of MMP-2 and MMP-9 were determined by western blot. ^#^*P* < 0.05 vs. Con (control group). ^∗^*P* < 0.05 vs. HG-stimulated group. Statistical significance was determined by one-way ANOVA.

TGF-β1 is an important regulatory factor during renal fibrosis, which can induce ECM accumulation (Yokoyama and Deckert, 1996). CTGF is a downstream factor of TGF-β1 (Li et al., 2012). MMPs are a family of proteolytic enzymes which can degrade ECM components and among MMPs, MMP-2, and MMP-9 have been implicated in the pathogenesis of DN (Sun et al., 2013). The results of qRT-PCR indicated that HG induced mRNA levels of TGF-β1 and CTGF in HBZY-1 cells and astilbin treatment inhibited HG-induced mRNA levels of TGF-β1 and CTGF (**Figures 4A,B**). The results of ELISA suggested that astilbin treatment exerted inhibitory effect on HG-induced secretion of TGF-β1 and CTGF in cell supernatant (**Figures 4C,D**). The results of western blot showed that astilbin treatment attenuated HG-induced decrease in expression of MMP-2 and MMP-9 (**Figure 4E**).

**FIGURE 5:**
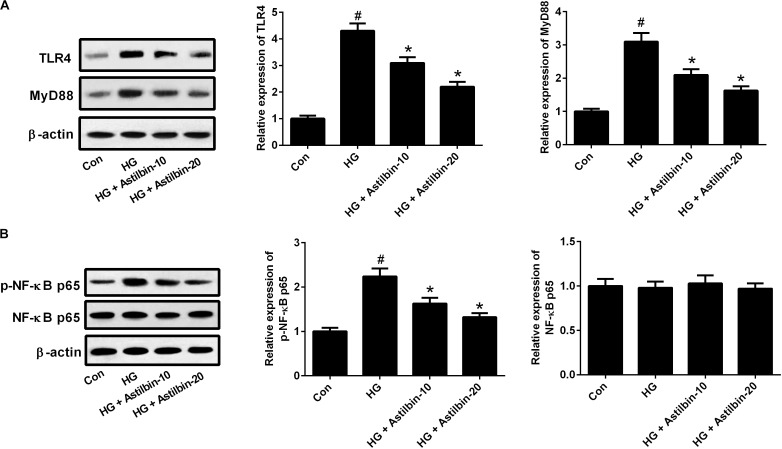
Effect of astilbin on HG-induced activation of the TLR4/MyD88 **(A)** and **(B)** NF-κB pathways. HBZY-1 cells were treated with 10 and 20 μg/ml of astilbin under HG condition for 24 h. The expression levels of TLR4, MyD88, p-NF-κB p65, and NF-κB p65 were measured by western blot. ^#^*P* < 0.05 vs. Con (control group). ^∗^*P* < 0.05 vs. HG-stimulated group. Statistical significance was determined by one-way ANOVA.

It has been reported that TLR4/MyD88 and NF-κB pathways are important pathways involved in inflammatory response (Wada and Makino, 2016). To evaluate whether the TLR4/MyD88 and NF-κB pathways were involved in the effect of HG induction, the expression levels of TLR4, MyD88, p-NF-κB p65, and NF-κB p65 were measured by western blot. The results in **Figure 5A** demonstrated that HG induced the expression levels of TLR4 and MyD88, and the induction was attenuated by astilbin treatment (10 and 20 μg/ml). The expression of p-NF-κB p65 was increased in the cells treated with HG, and astilbin treatment (10 and 20 μg/ml) inhibited the expression of p-NF-κB p65 (**Figure 5B**). The change of NF-κB p65 expression was not obvious in HBZY-1 cells treated with HG and/or astilbin.

### The TLR4/MyD88 and NF-κB Pathways Formed a Signaling Axis

**FIGURE 6:**
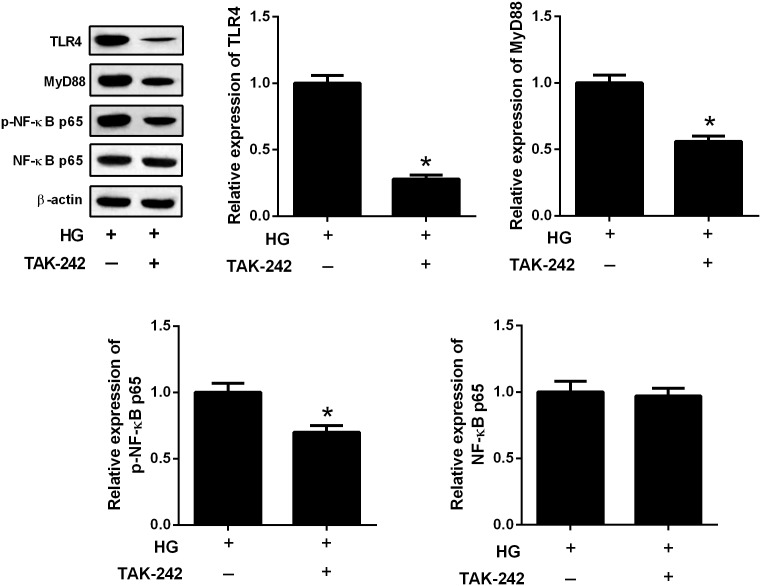
The TLR4/MyD88 and NF-κB pathways formed a signaling axis. HBZY-1 cells were treated with TAK-242 under HG condition for 24 h. The expression levels of TLR4, MyD88, p-NF-κB p65, and NF-κB p65 were measured by western blot. ^∗^*P* < 0.05. Statistical significance was determined by Student’s *t*-test.

To further evaluate the relation between TLR4/MyD88 and NF-κB pathways, HBZY-1 cells were treated with the inhibitor of TLR4 (TAK-242, 1 μM) for 24 h under HG condition. The expression levels of TLR4, MyD88, p-NF-κB p65, and NF-κB p65 were measured by western blot. The results in **Figure 6** showed that TAK-242 inhibited the expressions of TLR4, MyD88, and p-NF-κB p65 in the HBZY-1 cells under HG condition, indicating that the TLR4/MyD88 and NF-κB pathways formed a signaling axis.

### Inhibition of the TLR4/MyD88/NF-κB Pathway Suppressed HG-Induced Inflammatory Cytokine Production and ECM Accumulation

**FIGURE 7:**
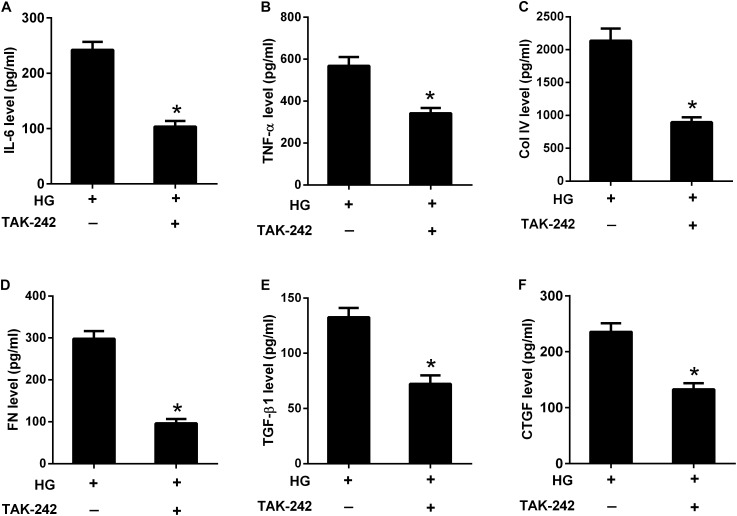
Inhibition of the TLR4/MyD88/NF-κB pathway suppressed HG-induced inflammation and ECM accumulation. HBZY-1 cells were treated with TAK-242 under HG condition for 24 h. The contents of IL-6 **(A)**, TNF-α **(B)**, Col IV **(C)**, FN **(D)**, TGF-β1 **(E)**, and CTGF **(F)** in cell supernatant were measured by ELISA. ^∗^*P* < 0.05. Statistical significance was determined by Student’s *t*-test.

To investigate the role of TLR4/MyD88/NF-κB pathway in the effect of HG, HBZY-1 cells were treated with the inhibitor of TLR4 (TAK-242, 1 μM) for 24 h under HG condition, and the contents of IL-6, TNF-α, Col IV, FN, TGF-β1, and CTGF in cell supernatant were measured by ELISA. As shown in **Figures 7A–F**, the contents of IL-6, TNF-α, Col IV, FN, TGF-β1, and CTGF were reduced in the cell supernatant after TAK-242 treatment, compared to cells without TAK-242 treatment. These results indicated that suppression of the TLR4/MyD88/NF-κB pathway inhibited HG-induced inflammatory cytokine production and ECM accumulation.

### TLR4 Overexpression Attenuated the Effect of Astilbin on HG-Induced Inflammatory Cytokine Production and ECM Accumulation

**FIGURE 8:**
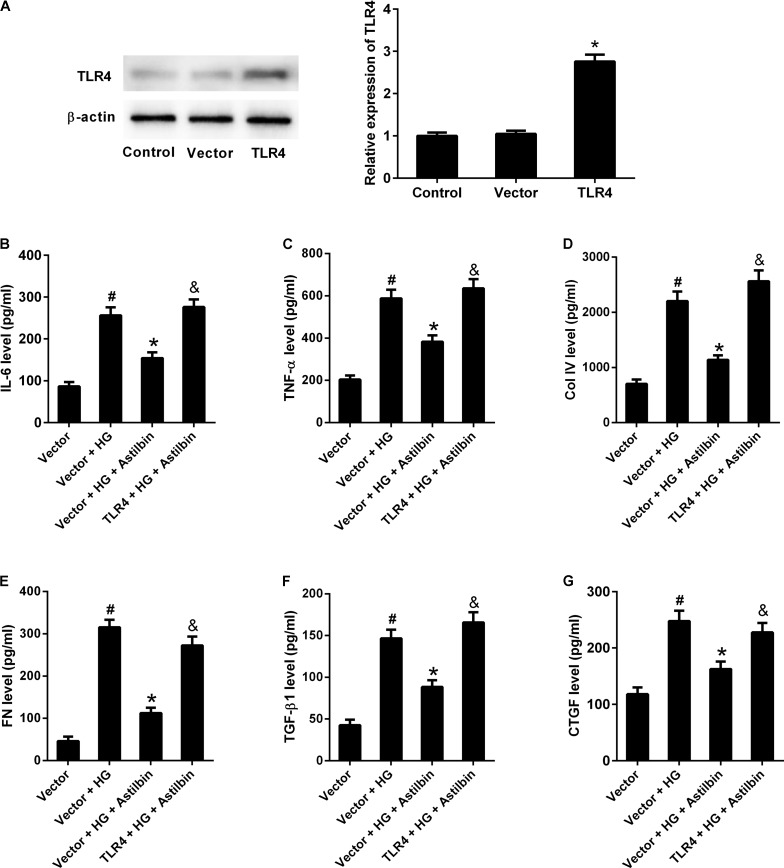
TLR4 overexpression attenuated the effect of astilbin on HG-induced inflammation and ECM accumulation. HBZY-1 cells were transfected with empty pcDNA3.1 vector (Vector) or pcDNA3.1-TLR4 (TLR4) for 24 h, and treated with 20 μg/ml of astilbin under HG condition for another 24 h. **(A)** Transfection efficiency was examined by western blot 48 h after transfection. The contents of IL-6 **(B)**, TNF-α **(C)**, Col IV **(D)**, FN **(E)**, TGF-β1 **(F)**, and CTGF **(G)** in cell supernatant were measured by ELISA. ^#^*P* < 0.05 vs. Vector group (cells were transfected with pcDNA3.1 vector). *P < 0.05 vs. Vector + HG group. ^&^*P* < 0.05 vs. Vector + HG + Astilbin group. Statistical significance was determined by one-way ANOVA.

To investigate whether TLR4 overexpression attenuated the effect of astilbin on HG-induced inflammatory cytokine production and ECM accumulation, HBZY-1 cells were transfected with empty pcDNA3.1 vector or pcDNA3.1-TLR4 for 24 h, and treated with 20 μg/ml of astilbin under HG condition for another 24 h. As shown in **Figure 8A**, TLR4 expression level was increased in cells transfected pcDNA3.1-TLR4. HG treatment increased the contents of IL-6, TNF-α, Col IV, FN, TGF-β1, and CTGF, and astilbin treatment exerted inhibitory effect on HG-induced secretion of IL-6, TNF-α, Col IV, FN, TGF-β1, and CTGF. However, TLR4 overexpression resisted the effect of astilbin (**Figures 8B–G**). These findings suggested that TLR4 overexpression attenuated the effect of astilbin on HG-induced inflammatory cytokine production and ECM accumulation.

## Discussion

The progression of DN consists of three steps: firstly, glomerular hypertrophy and hyperfiltration; secondarily, inflammation of glomeruli and tubulointerstitial regions; finally, accumulation of ECM and cell apoptosis (Makino et al., 1993). Previous studies have demonstrated that hyperglycaemia plays an important role in the pathogenesis of DN (Larkins and Dunlop, 1992; Feldt-Rasmussen, 2000). HG can promote the proliferation in glomerular mesangial cells (Jia et al., 2009). We also found that HG induced proliferation of HBZY-1 cells, and astilbin attenuated the induction. In recent years, many researchers have demonstrated that inflammation also plays an important role in the progression of DN (Lim and Tesch, 2012). Inflammation is characterized by increasing number of inflammatory cells and increasing expression levels of adhesion molecules, chemokines, and inflammatory cytokines (Barutta et al., 2015). Many researches focus on novel approaches targeting inflammation for the treatment of DN. In an *in vivo* study, combinations of Xiexin decoction constituents exhibited protective effect against DN by inhibiting the inflammation in rats (Wu et al., 2014). BAY 11-7082 protects rats from DN by reducing the expression of inflammatory cytokines including TNF-α, IL-1β, and IL-6, and inhibiting the oxidative damage mediated by hyperglycemia (Kolati et al., 2015). In the present study, we found that astilbin inhibited the production of HG-induced inflammatory cytokines including IL-1β and IL-6 in HBZY-1 cells, indicating that astilbin blocked HG-induced inflammation.

It has been shown that DN is characterized by excessive deposition of ECM components, which usually leads to glomerulosclerosis and tubulointerstitial fibrosis (Yokoyama and Deckert, 1996). The major components of ECM that have been found to be overexpressed in DN are Col I, Col III, Col IV, Col VI, FN, and laminin (Yokoyama and Deckert, 1996). Ma et al. (2017) reported that HG induced ECM accumulation including Col IV, laminin and FN in human mesangial cells. The expression levels of the ECM-associated molecules Col IV and FN in the supernatant of cells treated with HG were significantly increased (Zhou et al., 2018). In addition, TGF-β is a key cytokine for mediating both the induction and promotion of fibrogenesis and important for ECM accumulation in DN (Yokoyama and Deckert, 1996). Many studies have indicated that HG induced the secretion of TGF-β (Yokoyama and Deckert, 1996). TGF-β1 is a subtype of TGF-β and has been demonstrated to be induced by HG in human mesangial cells (Li et al., 2017). CTGF is a co-factor for TGF-β1 and is involved in the development of DN which is induced by HG, angiotensin II, and TGF-β1 (Chen et al., 2009). MMPs are a large family of Zn^2+^-dependent enzymes that degrade many ECM components (Sun et al., 2013). Among MMPs, MMP-2 and MMP-9 have been shown to be associated with DN, in which the expression levels of MMP-2 and MMP-9 were decreased (McLennan et al., 1994; Del Prete et al., 1997). We found that HG induced the expression of Col IV, FN, TGF-β1, CTGF, and ECM accumulation in HBZY-1 cells. The induction was attenuated by astilbin treatment. Astilbin treatment also attenuated HG-induced decrease in expression of MMP-2 and MMP-9. These data indicated that astilbin suppressed the HG-induced ECM accumulation in HBZY-1 cells.

TLR4 is a receptor of the innate immune system, and activation of the TLR4 pathways may cause chronic inflammation (Lucas and Maes, 2013). TLR4 pathways induce the production of reactive oxygen/nitrogen species and oxidative/nitrosative stress, leading to TLR-related diseases, including nephropathy, asthma, arteriosclerosis, stroke, type 2 diabetes, rheumatoid arthritis, and so on (Lucas and Maes, 2013). TLR4 has been reported to establish a link between inflammation and fibrosis in DN (Lepenies et al., 2011; Ma et al., 2014). NF-κB is a transcription factor family that is involved in various physiological processes, especially in inflammatory and immune responses (Pires et al., 2018). These evidences imply that inhibitors of these pathways may contribute to prevent the inflammatory diseases. The TLR4/MyD88 and NF-κB pathways are found to form a signaling axis and participate in inflammation responses (Lu et al., 2017; Qu et al., 2017; Zhang et al., 2017). It’s well established that TLR4 binding to adapter molecule MyD88 activates NF-κB and downstream signaling cascades, consequently leading to upregulation of multiple pro-inflammatory cytokines such as IL-6, TNF-α, and TGF-β1. These cytokines further promote to secrete collagen, which results in ECM (Liu et al., 2014, 2015). We also found that HG induced the activation of TLR4/MyD88 and NF-κB pathways, and the inhibitor of TLR4 inhibited the activation of NF-κB pathway, suggesting that TLR4/MyD88 and NF-κB pathways formed a signaling axis in HBZY-1 cells under HG condition. TAK-242, a small molecule specific inhibitor of TLR4 pathway, selectively binds to Cys747 of TLR4 and subsequently disrupts its interaction with adaptor molecules TIR domain-containing adaptor protein (TIRAP) and TRIF-related adaptor molecule (TRAM), thus suppressing TLR4 signal transduction and its downstream signaling events (Matsunaga et al., 2011). We found TAK-242 reversed the induction of HG on inflammatory responses and ECM accumulation, indicating that TLR4/MyD88/NF-κB pathway played an important role in HG-induced DN. However, the present study is restricted to *in vitro* studies, and an *in vivo* study is in progress.

In summary, this study demonstrated that HG induced cell proliferation and the production of IL-6, TNF-α, Col IV, FN, TGF-β1, and CTGF in HBZY-1 cells. The treatment of astilbin reversed the induction of HG. The TLR4/MyD88/NF-κB pathway was activated under HG condition and the inhibitor of TLR4 exhibited the same effect with astilbin on reversing the induction of HG. The results provided new sight that astilbin might be served as a therapy agent for DN.

## Author Contributions

FC designed the study and drafted the manuscript. FC and XZ performed the experiments. XZ, ZS, and YM analyzed the data. XZ and YM edited the manuscript. All authors approved the manuscript to be submitted.

## Conflict of Interest Statement

The authors declare that the research was conducted in the absence of any commercial or financial relationships that could be construed as a potential conflict of interest.
